# Prognostic factors in extensive-stage small cell lung cancer patients with organ-specific metastasis: unveiling commonalities and disparities

**DOI:** 10.1007/s00432-024-05621-9

**Published:** 2024-02-02

**Authors:** Yuanli Wu, Jing Zhang, Weiying Zhou, Zhongzhen Yuan, Hongmei Wang

**Affiliations:** 1 Department of Pharmacology, College of Pharmacy, Chongqing Medical University, Chongqing, China; 2 Department of Pathology, Chongqing University Cancer Hospital, Chongqing, China; 3 Department of Pharmacy, Chongqing University Cancer Hospital, No. 181, Hanyu Road, Shapingba District, Chongqing, China; 4 Department of Pharmacy, The First Affiliated Hospital of Chongqing Medical University, No. 1, Youyi RoadYuzhong District, YuanjiagangChongqing, 400016 China

**Keywords:** Small cell lung cancer, Metastasis, Survival, SEER, Nomogram

## Abstract

**Background:**

This study aimed to identify shared and distinct prognostic factors related to organ-specific metastases (liver, lung, bone, and brain) in extensive-stage small cell lung cancer (ES-SCLC) patients, then construct nomograms for survival prediction**.**

**Methods:**

Patient data for ES-SCLC were from the Surveillance, Epidemiology, and End Results (SEER) database from 2010 to 2019. Kaplan–Meier analysis was applied to estimate overall survival (OS), and Cox regression was used to identify prognostic factors. A Venn diagram was utilized to distinguish common and unique prognostic factors among the variables assessed**. **These identified prognostic factors were used to formulate a nomogram, and its predictive accuracy and reliability were evaluated using C-indexes, calibration curves, and receiver operating characteristic (ROC) curves.

**Results:**

A total of 24,507 patients diagnosed with ES-SCLC exhibiting metastases to the liver, lung, bone, and brain were included. The 6-month, 1-year, and 2-year OS rates were 46.1%, 19.7%, and 5.0%, respectively. Patients with liver metastasis demonstrated the most unfavorable prognosis, with a 1-year OS rate of 14.5%, while those with brain metastasis had a significantly better prognosis with a 1-year OS rate of 21.6%**. **The study identified seven common factors associated with a poor prognosis in ES-SCLC patients with organ-specific metastases: older age, male sex, unmarried status, higher T stage, presence of other metastases, and combination radiotherapy and chemotherapy. Furthermore, specific prognostic factors were identified for patients with metastasis to the liver, bone, and brain, including paired tumors, lack of surgical treatment at the primary site, and household income, respectively. To facilitate prognostic predictions, four nomograms were developed and subsequently validated. The performance of these nomograms was assessed using calibration curves, C-indexes, and the area under the curve (AUC), all of which consistently indicated good predictive accuracy and reliability.

**Conclusions:**

Patients diagnosed with ES-SCLC with organ-specific metastases revealed shared and distinct prognostic factors. The nomograms developed from these factors demonstrated good performance and can serve valuable clinical tools to predict the prognosis of ES-SCLC patients with organ-specific metastases.

**Supplementary Information:**

The online version contains supplementary material available at 10.1007/s00432-024-05621-9.

## Introduction

Lung cancer remains one of the most frequently diagnosed cancers and the leading cause of cancer-related deaths globally (Sung et al. 2021). Small cell lung cancer (SCLC) is categorized into two types: SCLC and non-small cell lung cancer (NSCLC). SCLC, a neuroendocrine tumor, constitutes approximately 14–17% of lung cancers, characterized by high aggressiveness, low differentiation, and high malignancy, with a 5-year survival rate of less than 7% (Tariq et al. 2021; Kahnert et al. 2016; Schwendenwein et al. 2021). About 80–85% of patients receive a diagnosis of advanced or extensive-stage SCLC (ES-SCLC) (Byers and Rudin 2015; Oronsky et al. 2022), leading to a low 2-year overall survival (OS) rate of 21.7% (Huang et al. 2021) and a shorter median OS compared to limited-stage SCLC patients (8.7 months vs. 16.9 months) (Demedts et al. 2010). Patients with ES-SCLC who show organ-specific metastases (such as liver, lung, bone, and brain) exhibit different survival outcomes. Early detection of similar and disparate prognostic factors related to organ-specific metastases in ES-SCLC patients is crucial to facilitate timely individualized treatment and improve the overall prognosis.

Several studies have explored prognostic factors related to organ-specific metastases in SCLC patients, but these investigations have certain limitations. Some studies suffer from restricted sample sizes and predominantly include cases before 2015 or 2016 (Reddy et al. 2020; Ren et al. 2016; Li et al. 2021). Furthermore, prognostic studies on SCLC with liver or lung metastases are scarce. Importantly, no studies have investigated common and distinct prognostic factors for liver, lung, bone, and brain metastasis in extensive-stage SCLC (ES-SCLC) patients. Nomograms, which integrate various predictors to provide comprehensive survival outcomes, have found widespread application in evaluating patient prognosis across different cancer types (Hu et al. 2022; Li et al. 2022). However, only two studies have so far involved constructing nomograms for the prognosis of ES-SCLC patients with metastasis, and the predictive performance of these models remains to be improved (Shan et al. 2021; Fan et al. 2021).

This study aimed to delineate common and distinct prognostic factors using a substantial cohort of ES-SCLC patients with organ-specific metastases (liver, lung, bone, and brain). Furthermore, a nomogram was formulated to forecast the prognosis of ES-SCLC patients, specifically with organ-specific metastases. This nomogram is a valuable reference tool for clinicians, guiding personalized treatment strategies for these patients.

## Material and methods

### Population

This study collected data on ES-SCLC patients from the open public database of the US National Cancer Institute and the Surveillance, Epidemiology, and End Results (SEER) Database. The inclusion criteria were patients diagnosed with ES-SCLC between 2010 and 2019 since data collection for metastatic sites, including bone and liver, began in 2010. Exclusions were individuals with unknown follow-up, primary tumor sites outside the lung, SCLC stages I to III, or those with unknown or no metastases. Limited-stage SCLC was defined as stages I to III (T any, N any, M0), while extensive-stage SCLC was defined as stage IV (T any, N any, M1a/b) (NCCN Website 2022). Ultimately, 24,507 ES-SCLC patients were included in this cohort analysis. Among them, 13,552, 5,787, 10,240, and 7,342 patients had liver, lung, bone, and brain metastases, respectively. Subsequently, they were randomly assigned to the training and validation cohorts in a 7:3 ratio. SEER*Stat version 8.4.0 was used to generate the patient list.

### Statistical analysis

Quantitative data, such as median OS, are presented as mean ± standard deviation. In contrast, categorical data, such as the proportion of males or females, are described as the number and percentage (*n*, %). The Kaplan–Meier method was used to estimate OS. Univariate and multivariate Cox regression analyses were used to identify potentially associated prognostic factors. In the results of univariate Cox regression, factors with *P* < 0.05 were included in the multivariate Cox regression analyses. Based on the multivariate Cox regression results, a Venn diagram was used to identify common and distinct prognostic factors in ES-SCLC patients with liver, lung, bone, and brain metastases. In this diagram, each set is represented by a circle, and the numbers at the intersections signify the shared prognostic factors within the data set (Bardou et al. 2014; Shen et al. 2022). Following the multivariate Cox regression analysis outcomes, predictive nomograms were developed for liver, lung, bone, and brain metastases. Subsequently, the performance of the nomogram was validated in an independent validation set.

The discriminatory and predictive capacities of the nomogram were assessed using C-indexes, calibration curves, and the area under the receiver operating characteristic (ROC) curve (AUC). Significance was established with a two-sided *P* value < 0.05. Statistical analysis was conducted using IBM SPSS Statistics version 23.0 software. The nomograms, ROC curves, and calibration curves were generated using the R software (version 4.2.2).

## Results

### Clinicopathological characteristics

**Fig. 1:**
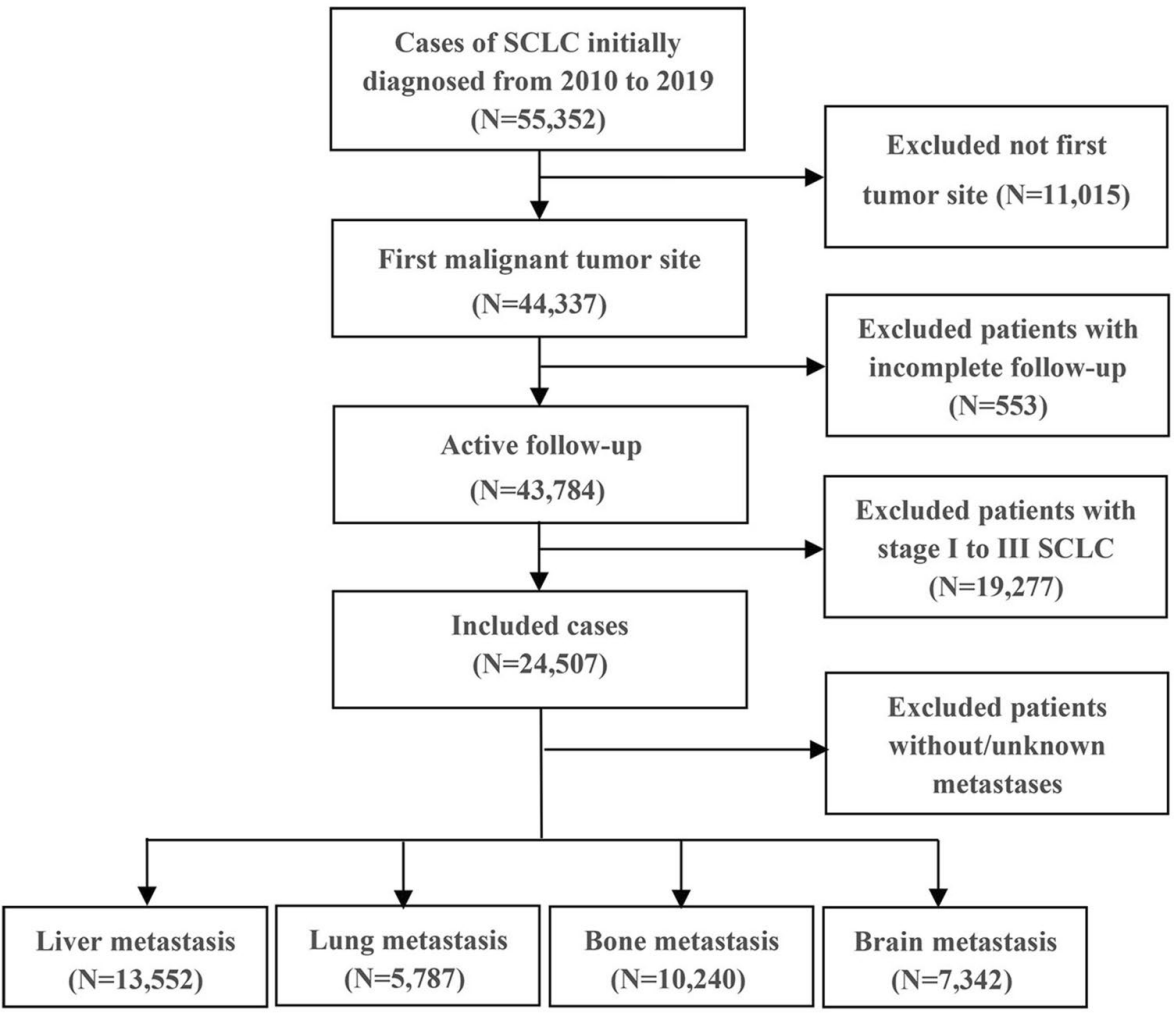
Flow chart of the selection of EC-SCLC patients

A total of 24,507 patients with organ-specific metastases were included. The detailed patient selection workflow is shown in Fig. 1. Among these patients, 13,552 had liver metastasis, 5787 had lung metastasis, 10,240 had bone metastasis, and 7342 had brain metastasis. Individuals with distant metastases were more likely to be older (> 60 years, 77.2%, *n* = 18,931), male (52.6%, *n* = 12,882), and unmarried (48.1%, *n* = 11,787). Most patients were of white ethnicity (87.3%, *n* = 21,384), and 55.0% (*n* = 13,482) had a household income exceeding $60,000 per year. Regarding cancer characteristics, 43.9% (*n* = 10,751) of the cancers were in the upper lobe, 10.3% (*n* = 2,536) were grade IV stage, 35.3% (*n* = 8,663) were T4 stage, and 51.2% (*n* = 12,545) were N2 stage. More than half of the patients (52.0%, *n* = 12,732) had tumors in the right lung. Regarding treatment, only 0.7% (*n* = 166) of the patients underwent surgical treatments at the primary site. Less than half of the patients (37.8%, *n* = 9,273) received radiotherapy, while the majority (66.0%, *n* = 16,177) underwent chemotherapy. Table 1 provides a comprehensive overview of the clinical and tumor characteristics of the training and validation cohort. The baseline characteristics of training and validation cohort are balanced.

**Table 1:** Clinical and tumor characteristics of patients diagnosed with ES-SCLC

Subject characteristics	Total distant metastases (*N* = 24,507)	Liver metastasis (*N* = 13,552)		Lung metastasis (*N* = 5787)		Bone metastasis (*N* = 10,240)		Brain metastasis (*N* = 7342)	
		Training cohort (*N* = 9500, 70.1%)	Validation cohort (*N* = 4052, 29.9%)	Training cohort (*N* = 4070, 70.3%)	Validation cohort (*N* = 1717, 29.7%)	Training cohort (*N* = 7146, 69.8%)	Validation cohort (*N* = 3094, 30.2%)	Training cohort (*N* = 5125, 69.8%)	Validation cohort (*N* = 2217, 30.2%)
*Age* (*years*)									
≤ 50	792 (3.2)	308 (3.2)	127 (3.1)	113 (2.8)	50 (2.9)	250 (3.5)	97 (3.1)	188 (3.7)	99 (4.5)
51–60	4784 (19.5)	1813 (19.1)	763 (18.8)	725 (17.8)	271 (15.8)	1456 (20.4)	641 (20.7)	1206 (23.5)	509 (23.0)
61–70	8990 (36.7)	3432 (36.1)	1511 (37.3)	1438 (35.3)	599 (34.9)	2737 (38.3)	1171 (37.8)	2009 (39.2)	888 (40.1)
71–80	7299 (29.8)	2913 (30.7)	119 9(29.6)	1288 (31.6)	555 (32.3)	2090 (29.2)	874 (28.2)	1385 (27.0)	556 (25.1)
≥ 81–90	2642 (10.8)	1034 (10.9)	452 (11.2)	506 (12.4)	242 (14.1)	613 (8.6)	311 (10.1)	337 (6.6)	165 (7.4)
*Sex*									
Female	11,625 (47.4)	4455 (46.9)	1936 (47.8)	1925 (47.3)	811 (47.2)	3215 (45.0)	1387 (44.8)	2459 (48.0)	1050 (47.4)
Male	12,882 (52.6)	5045 (53.1)	2116 (52.2)	2145 (52.7)	906 (52.8)	3931 (55.0)	1707 (55.2)	2666 (52.0)	1167 (52.6)
*Race*									
White	21,384 (87.3)	8452 (89.0)	3608 (89.0)	3505 (86.1)	1475 (85.9)	6340 (88.7)	2746 (88.8)	4330 (84.5)	1896 (85.5)
Black	2023 (8.3)	688 (7.2)	273 (6.7)	363 (8.9)	147 (8.6)	524 (7.3)	223 (7.2)	515 (10.0)	212 (9.6)
Others^a^	1059 (4.3)	347 (3.7)	162 (4.0)	197 (4.8)	93 (5.4)	277 (3.9)	119 (3.8)	272 (5.3)	104 (4.7)
Unknown	41 (0.2)	13 (0.1)	9 (0.2)	5 (0.1)	2 (0.1)	5 (0.1)	6 (0.2)	8 (0.2)	5 (0.2)
*Marital status*									
Married	11,787 (48.1)	4636 (48.8)	1950 (48.1)	1885 (46.3)	800 (46.6)	3556 (49.8)	1573 (50.8)	2535 (49.5)	1084 (48.9)
Unmarried^b^	11,698 (47.7)	4494 (47.3)	1927 (47.6)	2024 (49.7)	841 (49.0)	3323 (46.5)	1403 (45.3)	2401 (46.8)	1049 (47.3)
Unknown	1022 (4.2)	370 (3.9)	175 (4.3)	161 (4.0)	76 (4.4)	267 (3.7)	118 (3.8)	189 (3.7)	84 (3.8)
*Household income*									
≤ 60,000$	11,025 (45.0)	4295 (45.2)	1804 (44.5)	1814 (44.6)	754 (43.9)	3244 (45.4)	1370 (44.3)	2251 (43.9)	1020 (46)
> 60,000$	13,482 (55.0)	5205 (54.8)	2248 (55.5)	2256 (55.4)	963 (56.1)	3902 (54.6)	1724 (55.7)	2874 (56.1)	1197 (54)
*Site*									
Main bronchus	2661 (10.9)	1085 (11.4)	429 (10.6)	464 (11.4)	180 (10.5)	792 (11.1)	370 (12.0)	529 (10.3)	225 (10.1)
Upper lobe	10,751 (43.9)	4056 (42.7)	1717 (42.4)	1737 (42.7)	748 (43.6)	3212 (44.9)	1380 (44.6)	2381 (46.5)	1045 (47.1)
Middle lobe	854 (3.5)	304 (3.2)	144 (3.6)	125 (3.1)	38 (2.2)	241 (3.4)	104 (3.4)	189 (3.7)	86 (3.9)
Lower lobe	4924 (20.1)	1970 (20.7)	846 (20.9)	768 (18.9)	319 (18.6)	1444 (20.2)	626 (20.2)	1059 (20.7)	437 (19.7)
Overlapping	386 (1.6)	134 (1.4)	68 (1.7)	67 (1.6)	31 (1.8)	115 (1.6)	63 (2.0)	63 (1.2)	33 (1.5)
Unknown	4931 (20.1)	1951 (20.5)	848 (20.9)	909 (22.3)	401 (23.4)	1342 (18.8)	551 (17.8)	904 (17.6)	391 (17.6)
*Histological grade*									
Grade I	31 (0.1)	8 (0.1)	2 (0)	4 (0.1)	4 (0.2)	8 (0.1)	5 (0.2)	6 (0.1)	4 (0.2)
Grade II	48 (0.2)	14 (0.2)	1 (0)	11 (0.3)	1 (0.1)	9 (0.1)	6 (0.2)	15 (0.3)	3 (0.1)
Grade III	1912 (7.8)	666 (7.0)	289 (7.1)	356 (8.7)	146 (8.5)	549 (7.7)	268 (8.7)	434 (8.5)	205 (9.2)
Grade IV	2536 (10.3)	891 (9.4)	388 (9.6)	430 (10.6)	189 (11.0)	726 (10.2)	301 (9.7)	525 (10.2)	246 (11.1)
Unknown	19,980 (81.5)	7921 (83.4)	3372 (83.2)	3269 (80.3)	1377 (80.2)	5854 (81.9)	2514 (81.3)	4145 (80.9)	1759 (79.3)
*Laterality*									
Right	12,732 (52.0)	4910 (51.7)	2103 (51.9)	2053 (50.4)	882 (51.4)	3714 (52.0)	1634 (52.8)	2661 (51.9)	1154 (52.1)
Left	9691 (39.5)	3735 (39.3)	1566 (38.6)	1583 (38.9)	646 (37.6)	2892 (40.5)	1219 (39.4)	2071 (40.4)	886 (40.0)
Paired	1514 (6.2)	629 (6.62)	282 (7.0)	216 (5.3)	110 (6.4)	384 (5.4)	168 (5.4)	285 (5.6)	137 (6.2)
Bilateral	344 (1.4)	136 (1.4)	56 (1.4)	172 (4.2)	64 (3.7)	87 (1.2)	40 (1.3)	56 (1.1)	24 (1.1)
Other	226 (0.9)	90 (1.0)	45 (1.1)	46 (1.1)	15 (0.9)	69 (1.0)	33 (1.1)	52 (1.0)	16 (0.7)
*Lymphatic metastasis*									
N0	2800 (11.4)	989 (10.4)	414 (10.2)	386 (9.5)	172 (10.0)	631 (8.8)	248 (8.0)	674 (13.2)	312 (14.1)
N1	1623 (6.6)	608 (6.4)	261 (6.4)	259 (6.4)	86 (5.0)	432 (6.0)	180 (5.8)	423 (8.3)	167 (7.5)
N2	12,545 (51.2)	5047 (53.1)	2141 (52.8)	2006 (49.3)	858 (50.0)	3655 (51.1)	1624 (52.5)	2517 (49.1)	1072 (48.4)
N3	5858 (23.9)	2175 (22.9)	944 (23.3)	1208 (29.7)	512 (29.8)	2032 (28.4)	879 (28.4)	1200 (23.4)	521 (23.5)
Unknown	1681 (6.9)	681 (7.2)	292 (7.2)	211 (5.2)	89 (5.2)	396 (5.5)	163 (5.3)	311 (6.1)	145 (6.5)
T1	2095 (8.5)	802 (8.4)	352 (8.7)	160 (3.9)	62 (3.6)	611 (8.6)	241 (7.8)	528 (10.3)	211 (9.5)
T2	4844 (19.8)	1917 (20.2)	840 (20.7)	466 (11.4)	178 (10.4)	1407 (19.7)	646 (20.9)	1044 (20.4)	452 (20.4)
T3	4534 (18.5)	1723 (18.1)	731 (18.0)	898 (22.1)	392 (22.8)	1374 (19.2)	567 (18.3)	908 (17.7)	412 (18.6)
T4	8663 (35.3)	3219 (33.9)	1335 (32.9)	2129 (52.3)	895 (52.1)	2600 (36.4)	1139 (36.8)	1799 (35.1)	799 (36.0)
Unknown	4371 (17.8)	1839 (19.4)	794 (19.6)	417 (10.2)	190 (11.1)	1154 (16.1)	501 (16.2)	846 (16.5)	343 (15.5)
*Liver metastasis*									
None	10,282 (42.0)	–	–	2121 (52.1)	894 (52.1)	3082 (43.1)	1330 (43.0)	3363 (65.6)	1463 (66.0)
Yes	13,552 (55.3)	–	–	1860 (45.7)	781 (45.5)	3956 (55.4)	1701 (55.0)	1666 (32.5)	713 (32.2)
Unknown	673 (2.7)	–	–	89 (2.2)	42 (2.4)	108 (1.5)	63 (2.0)	96 (1.9)	41 (1.8)
*Lung metastasis*	13,552 (45.7)								
None	17,609 (71.9)	7292 (76.8)	3114 (76.9)	–	–	5433 (76.0)	2398 (77.5)	4048 (79.0)	1751 (79.0)
Yes	5787 (23.6)	1846 (19.4)	795 (19.6)	–	–	1464 (20.5)	594 (19.2)	912 (17.8)	394 (17.8)
Unknown	1111 (4.5)	362 (3.8)	143 (3.5)	–	–	249 (3.5)	102 (3.3)	165 (3.2)	72(3.2)
*Bone metastasis*									
None	13,473 (55.0)	5305 (55.8)	2285 (56.4)	2537 (62.3)	1042 (60.7)	–	–	3553 (69.3)	1517 (68.4)
Yes	10,240 (41.8)	3972 (41.8)	1685 (41.6)	1423 (35.0)	635 (37.0)	–	–	1501 (29.3)	653 (29.5)
Unknown	794 (3.2)	223 (2.4)	82 (2.0)	110 (2.7)	40 (2.3)	–	–	71 (1.4)	47 (2.1)
*Brain metastasis*									
None	16,281 (66.4)	7567 (79.7)	3223 (79.5)	3046 (74.8)	1257 (73.2)	5459 (76.4)	2364 (76.4)	–	–
Yes	7342 (30.0)	1658 (17.4)	721 (17.8)	895 (22.0)	411 (23.9)	1501 (21.0)	653 (21.1)	–	–
Unknown	884 (3.6)	275 (2.9)	108 (2.7)	129 (3.2)	49 (2.9)	186 (2.6)	77 (2.5)	–	–
*Surg(pri)*									
Yes	166 (0.7)	40 (0.4)	19 (0.5)	27 (0.7)	8 (0.5)	36 (0.5)	11 (0.4)	47 (0.9)	19 (0.9)
None	24,341 (99.3)	9460 (99.5)	4033 (99.5)	4043 (99.3)	1709 (99.5)	7110 (99.5)	3083 (99.6)	5078 (99.1)	2198 (99.1)
*Radiation*									
Yes	9273 (37.8)	2517 (26.5)	1123 (27.7)	1340 (32.9)	583 (34.0)	2816 (39.4)	1215 (39.3)	3528 (68.8)	1510 (68.1)
No/unknown	15,234 (62.2)	6983 (73.5)	2929 (72.3)	2730 (67.1)	1134 (66.0)	4330 (60.6)	1879 (60.7)	1597 (31.2)	707 (31.9)
*Chemotherapy*									
Yes	16,177 (66.0)	6046 (63.6)	2595 (64.0)	2603 (64.0)	1093 (63.7)	2816 (39.4)	2227 (72.0)	3562 (69.5)	1527 (68.9)
No/unknown	8330 (34.0)	3454 (36.4)	1457 (36.0)	1467 (36.0)	624 (36.3)	4330 (60.6)	867 (28.0)	1563 (30.5)	690 (31.1)

Surg(pri) = surgical treatments of primary site

^a^Includes American Indian/Alaska Native and Asian or Pacific Islander

^b^Includes single, separated, widowed, and divorced

### Survival outcomes in patients with organ-specific metastases

The 6-month, 1-year, and 2-year OS rates were 46.1%, 19.7%, and 5.0%, respectively, for all patients with organ-specific metastases. The mean OS for these patients was 8.0 [95% confidence interval (CI) 7.8–8.1] months, with a median OS of 5.0 [95% CI 4.86–5.14] months. Mean OS was reduced in patients with distant metastases to the brain (8.7 [95% CI 8.4–9.1] months), lung (7.9 [95% CI 7.5–8.3] months), bone (7.7 [95% CI 7.4–8.0] months), and liver (6.0 [95% CI 5.8–6.2] months). The median OS for liver and lung metastasis patients was 3.0 [95% CI 2.8–3.2] months and 4.0 [95% CI 3.7–4.3] months, respectively. Patients with bone or brain metastases had a similar median OS 5.0 [95% CI 4.8–5.2] months. Kaplan–Meier curves of OS in ES-SCLC patients with different distant metastasis were shown in Fig. S1. Regarding the survival rate, the 6-month OS exceeded 40% in patients with liver metastasis (41.1%), lung metastasis (43.9%), bone metastasis (49.3%), and brain metastasis (47.8%). Patients with brain metastases had the highest 1-year OS rate (21.6%), followed by those with lung metastasis (19.0%), bone metastasis (18.9%), and liver metastasis (14.5%). The 2-year OS rates for patients with brain metastasis and lung metastasis were 5.7% and 5.3%, respectively, surpassing those for patients with bone metastasis (4.1%) and liver metastasis (2.4%). Overall, patients with liver metastases had the poorest prognosis in terms of one-year and two-year survival, while patients with brain metastases exhibited the most favorable prognosis.

### Prognostic factors in ES-SCLC patients with organ-specific metastases

Univariate Cox regression analysis revealed that, except for two factors (household income and histological grade) for liver metastasis, four factors (household income, histological grade, N stage, and surgical treatments of the primary site) for lung metastasis, three factors (race, household income, and histological grade) for bone metastasis, and three factors (race, site, and histological grade) for brain metastasis, all other factors were correlated with the prognosis of ES-SCLC patients. Multivariate Cox analysis showed that independent prognostic factors for ES-SCLC patients with liver metastases included age, sex, race, marital status, laterality, T stage, lung metastasis, bone metastasis, brain metastasis, radiotherapy, and chemotherapy. For those with lung metastases, independent prognostic factors were age, sex, race, marital status, T stage, liver metastasis, bone metastasis, brain metastasis, radiotherapy, and chemotherapy. In the case of bone metastasis, independent prognostic factors were age, sex, marital status, N stage, T stage, liver metastasis, lung metastasis, brain metastasis, surgical treatments of the primary site, radiotherapy, and chemotherapy. Lastly, independent prognostic factors for ES-SCLC patients with brain metastasis included age, sex, marital status, household income, stage N, stage T, liver metastasis, lung metastasis, bone metastasis, radiotherapy, and chemotherapy. Detailed information on associated prognostic factors and their hazard ratios with 95% CIs is presented in Tables S1–S4.

**Fig. 2:**
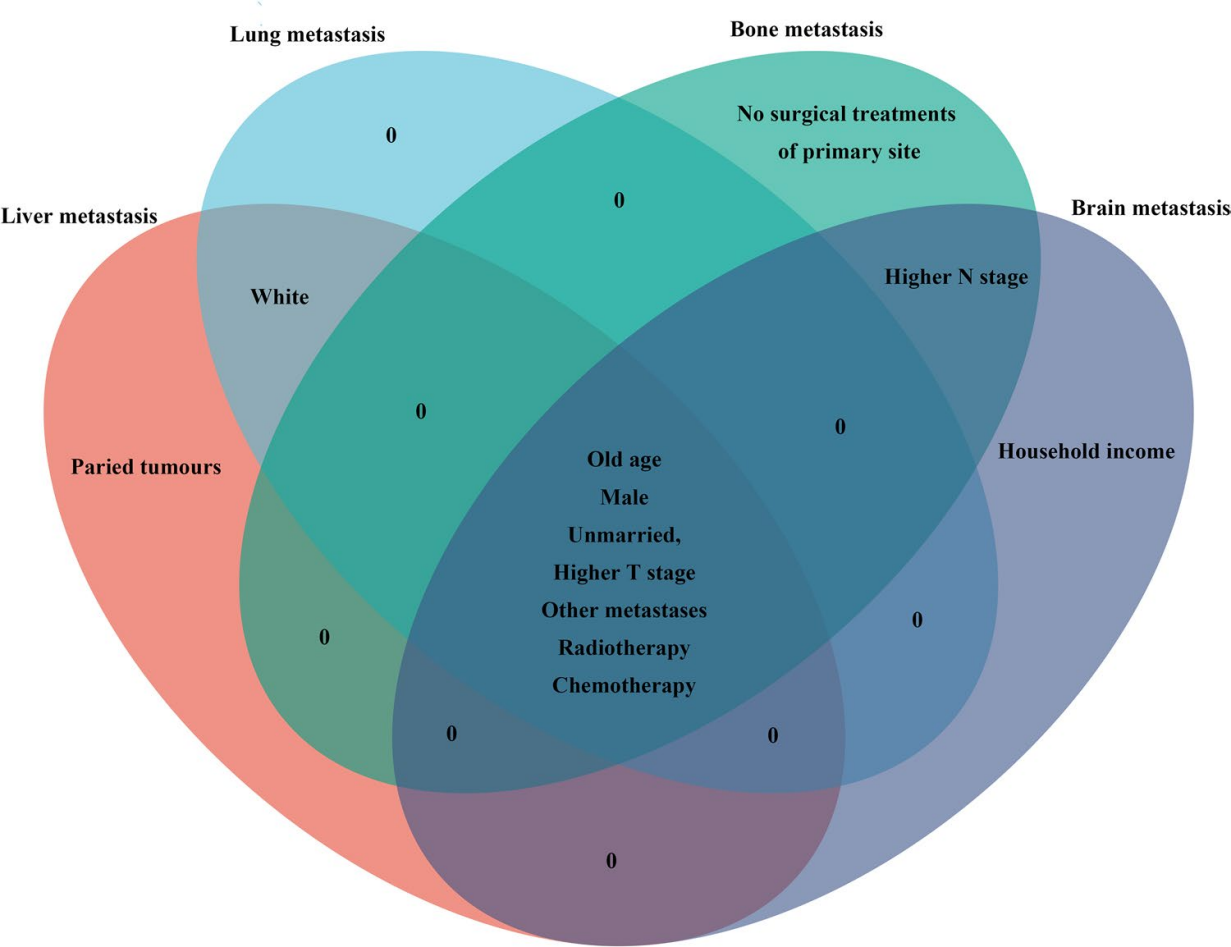
Common and distinct prognostic factors identified by the Venn diagram in ES-SCLC patients with organ-specific metastases (liver, lung, bone, and brain). The intersecting parts of the circles represent the common prognostic factors. Zero (0) represents no common or distinct factors

Common and distinct prognostic factors were identified in ES-SCLC patients with metastases to the liver, lung, bone, and brain using a Venn diagram (Fig. 2). A total of seven common factors, namely old age, male sex, unmarried status, higher T stage, presence of other metastases, and receipt of radiotherapy and chemotherapy, were positively correlated with a poorer prognosis in ES-SCLC patients with metastases to the liver, lung, bone, and brain. Higher N stage was the only shared prognostic factor among bone and brain metastasis patients. The white race emerged as the singular common prognostic factor between patients with liver and lung metastasis. In particular, certain specific prognostic factors were identified in patients with different metastases. Paired tumors (also called midline tumors), the absence of surgical treatment at the primary site, and household income were unique prognostic factors for ES-SCLC patients with metastasis to the liver, bone, and brain, respectively.

### Performance of the prognostic and predictive nomograms

**Fig. 3:**
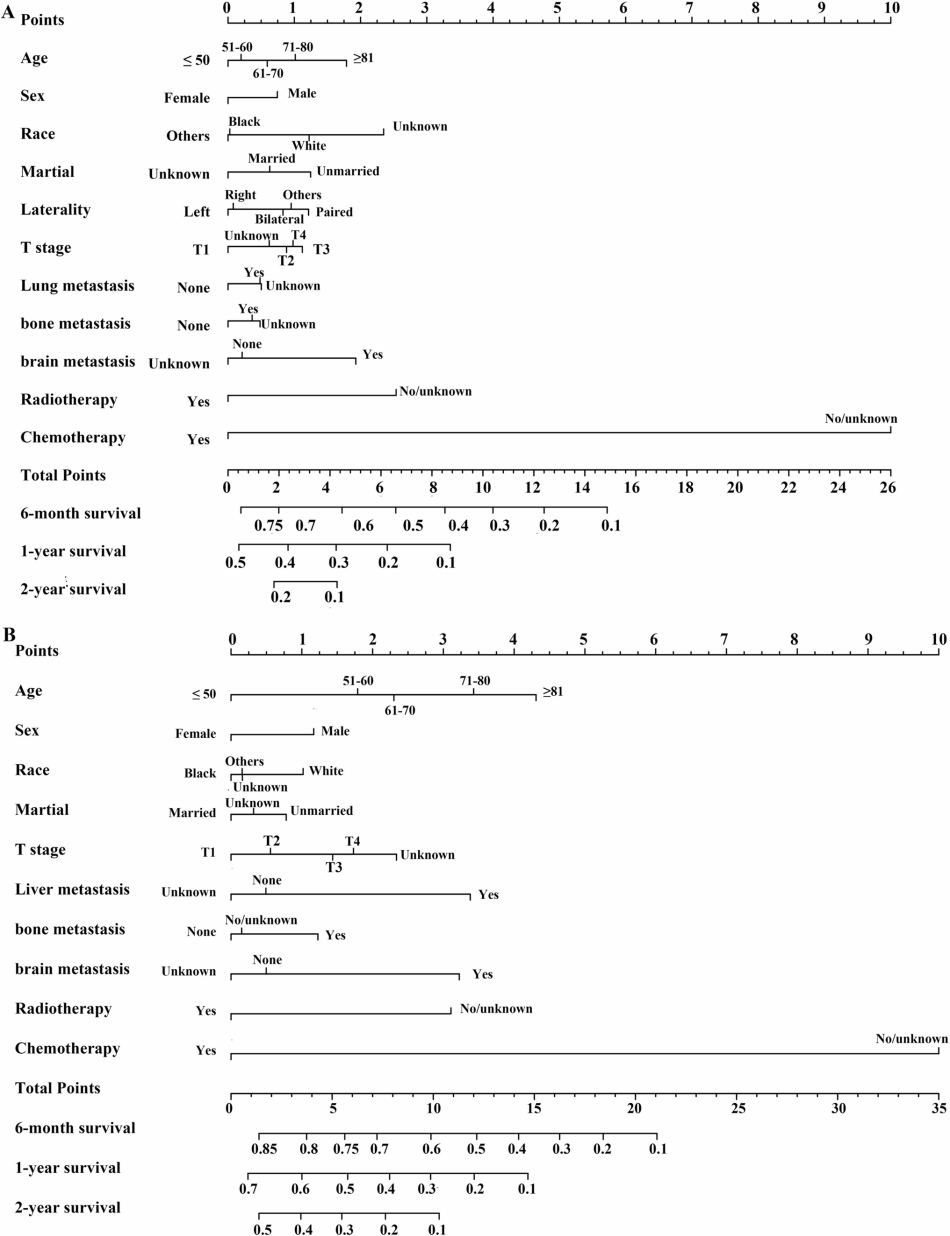
Nomogram to predict the 6-month, 1-year, and 2-year OS rates of ES-SCLC patients with liver metastasis (**A**), lung metastasis (**B**)

**Fig. 4:**
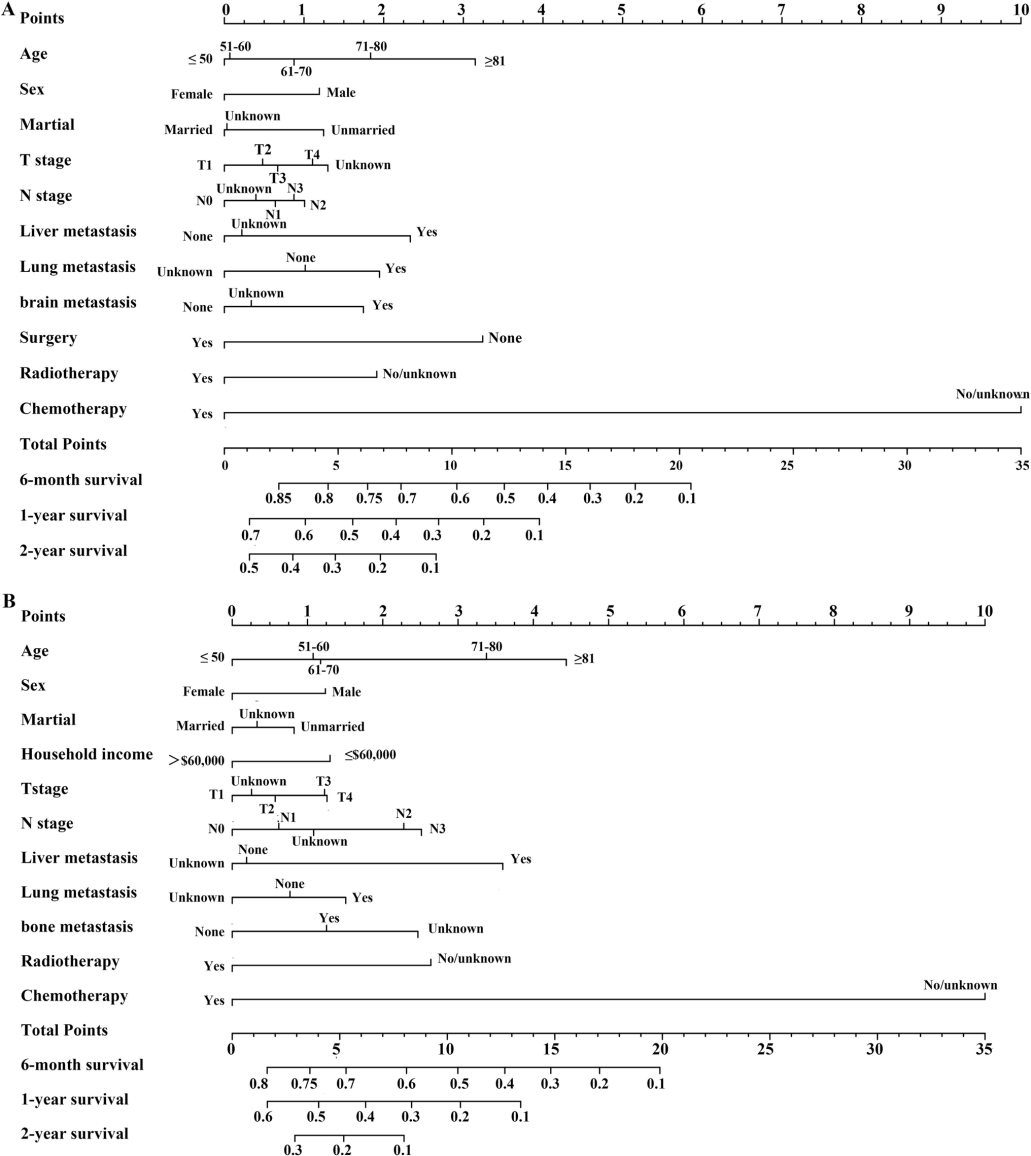
Nomogram to predict the 6-month, 1-year, and 2-year OS rates of ES-SCLC patients with bone metastasis (**A**), and brain metastasis (**B**)

All significant variables related to the prognosis of ES-SCLC patients with liver, lung, bone, and brain metastases were incorporated into the nomogram to predict the 6-month, 1-year, and 2-year OS rates (Figs. 3A–B and 4A–B). The C-indices to predict OS in patients with liver metastasis, lung metastasis, bone metastasis, and brain metastasis were 0.746 (95% CI 0.732–0.758), 0.749 (95% CI 0.731–0.767), 0.730 (95% CI 0.716–0.744), and 0.727 (95% CI 0.711–0.743), respectively, in the training cohort and 0.746 (95% CI 0.728–0.764), 0.762 (95% CI 0.735–0.789), 0.726 (95% CI 0.704–0.748), and 0.735 (95% CI 0.710–0.760), respectively, in the validation cohort. In ES-SCLC patients with liver metastasis, the calibration curves for the training cohort (Fig. S2A–C) and the validation cohort (Fig. S2D–F) demonstrated good agreement between predicted and observed survival probabilities. Similar results were observed for the calibration curves in patients with lung metastasis, bone metastasis, and brain metastasis, and the calibration curves are shown in Figs. S3, S4, and S5, respectively.

The AUC for the 6-month, 1-year, and 2-year OS rates of patients with liver metastases in the training cohort was 0.815, 0.735, and 0.675, respectively (Fig. S2G–I) and 0.823, 0.731, and 0.726, respectively, in the validation cohort (Fig. S2J–L). The AUCs of the nomogram for the 6-month, 1-year, and 2-year OS rates of patients with lung metastasis reached 0.822, 0.766, and 0.726, respectively (Fig. S3G–I) in the training cohort and 0.839, 0.797, and 0.769 in the validation cohort, respectively (Fig. S3 J–L). The AUC for the 6-month, 1-year, and 2-year survival of patients with bone metastasis reached 0.794, 0.733, and 0.677, respectively (Fig. S4G–I), and 0.797, 0.734, and 0.680 in the validation cohort, respectively (Fig. S4J–L). AUCs for the 6-month, 1-year, and 2-year OS of patients with brain metastasis in the training cohort were 0.786, 0.750, and 0.693, respectively (Fig. S5G–I) and 0.800, 0.737, and 0.681 in the validation cohort, respectively (Fig. S5J–L).

## Discussion

In this study, the predominant metastasis sites identified were the liver, followed by bone, brain, and lung. This is consistent with the findings of previous studies that included 251 and 7481 SCLC patients, respectively (Li et al. 2021; Shan et al. 2021). Through this retrospective study with a large sample, we not only delineated survival disparities in ES-SCLC patients but also identified common and distinct prognostic factors in cases of organ-specific metastases, ultimately constructing predictive models. We observed a rapid decline in OS rate among ES-SCLC patients with organ-specific metastasis, with 6-month, 1-year, and 2-year OS rates ranging from 41.1% to 49.3%, 14.5% to 21.6%, and 2.4% to 5.7%, respectively. ES-SCLC patients exhibited the highest 6-month OS rates (> 40%) but a low 2-year OS rate, regardless of metastatic locations in the liver, lung, bone, or brain. In particular, previous studies predominantly emphasized 1- or 2-year OS rates, with limited focus on 6-month OS in ES-SCLC patients. The 6-month OS rate for ES-SCLC patients, as evidenced in our study, remains significantly shorter than that of NSCLC patients.

Regarding 1-year OS, our study revealed that patients with liver metastasis exhibited the shortest 1-year OS (14.5%), while patients with brain metastasis (21.6%), bone metastasis (18.9%), and lung metastasis (19.0%) showed slightly longer OS rates. The results are consistent with a previous study (Ren et al. 2016). However, another study reported higher 1-year OS rates than observed in our study, including 18.8% for liver metastases, 40.9% for brain metastases, 25.0% for bone metastases, and 33.4% for lung metastases(Nakazawa et al. 2012). This discrepancy may be due to the fact that this study only focused on patients with sole specific organ metastasis, whereas our study included both patients with sole and multiple organ metastasis. Limited studies have reported 2-year OS rates. In one study, the 2-year OS rate for ES-SCLC was 2.9% for patients with liver metastasis, 6.4% for patients with bone metastasis, and 7.9% for patients with brain metastasis, similar to our results (Ren et al. 2016). Although our findings may not fully align with previous studies, we confirmed a consistent trend in which OS rates were the poorest for patients with liver metastases and comparatively better for those with brain metastases (Nakazawa et al. 2012).

Limited studies have investigated prognostic factors in metastasis to different organs in ES-SCLC patients. Only two studies have reported some prognostic factors for brain metastasis in ES-SCLC patients (Reddy et al. 2020; Shan et al. 2021), and the identification of prognostic factors in our study did not fully align with these studies. For example, we found that race was not a prognostic factor for brain metastasis and that neither study clarified the impact of treatments such as radiotherapy and chemotherapy on patient prognosis. Additionally, our study revealed that patients with a household income ≤ $60,000 had a worse prognosis, a result not reported in any previous study. The poorer prognosis in this income group may be attributed to financial constraints, limiting patient access to follow-up treatment and ultimately leading to shorter survival times.

Regarding prognostic factors for bone metastasis, our study found that age, sex, liver metastasis, brain metastasis, radiotherapy, and chemotherapy were independent prognostic factors consistent with a previous study (Fan et al. 2021). However, we found that the primary site was not a prognostic factor in patients with bone metastases, which diverged from the findings of that study. Furthermore, we identified surgical treatments of primary tumor, lung metastasis, N stage, and T stage as independent prognostic factors in ES-SCLC patients with bone metastasis, none of which has previously been reported.

Relevant studies have not reported prognostic factors in patients with liver and lung metastases. The prognostic factors identified in this study could prove valuable, offering clinical practitioners a more comprehensive understanding of the prognosis of ES-SCLC patients with metastasis. Early identification of common and distinct prognostic factors in ES-SCLC patients with organ-specific metastases is crucial to facilitate timely individualized treatment and improve prognosis. In this study, we identified seven common factors. Three additional prognostic factors, paired tumors/right-sided tumors, lack of surgical treatment at the primary site, and household income (≤ $60,000/ > $60,000), were unique for ES-SCLC patients with liver, bone, and brain metastases, respectively. The impact of paired tumors compared to right-sided tumors on the prognosis of patients with liver metastases only, and not those with other metastases, may be due to the likelihood that SCLC patients with paired tumors are more prone to have distant multiple metastases.

Previous reports indicated that patients with distant multiple metastases generally had poorer survival than those with a single metastasis (Fan et al. 2021; Cai et al. 2018). However, more research is needed to validate this hypothesis. Furthermore, while previous studies concluded that lack of surgical treatment at the primary site did not influence the prognosis of patients with bone metastasis, our study identified this factor as a specific prognostic determinant in patients with bone metastasis. This discrepancy may be attributed to variations in sample sizes between the two studies, leading to inconsistent results. The influence of household income on the prognosis of patients with brain metastases alone could be explained by the fact that patients with brain metastases typically require both radiotherapy and chemotherapy, increasing the overall treatment cost compared to patients with other metastases. Therefore, family income is a unique prognostic factor in patients with brain metastases. The common and specific prognostic factors identified in our study have not previously been reported. Although these factors require further validation in additional studies, we recommend early screening for patients with these factors. In addition, patients with bone metastasis can be considered for surgical treatment at the primary site, as such an intervention could potentially contribute to prolonged survival.

Nomograms, which integrate various prognostic and determinant variables, have become valuable prognostic tools in oncology and medicine (Balachandran et al. 2015). We constructed four predictive nomograms according to the common and specific prognostic factors identified in this study. The C-index and the AUCs in this study indicates that the nomograms exhibited good predictive performance in predicting OS in ES-SCLC patients with specific organ metastases. However, our results revealed that the AUCs of the nomograms for predicting 6-month and 1-year survival were higher than those for predicting 2-year survival. In other words, the nomograms demonstrated better predictive performance for 6-month and 1-year survival in ES-SCLC patients, while further refinement is needed to predict 2-year survival. However, the prognostic performance of our nomograms exceeded that observed in a previous study (Shan et al. 2021). Moreover, one previous study developed a nomogram for metastatic patients based on cases from 2010 to 2016 (Gao et al. 2020). However, this model included limited factors and did not assess the predictive performance of the nomograms at 6-month, 1-year and 2-year, separately. This study has several limitations. First, the population was from the SEER database, and due to its limitations, information on certain crucial prognostic factors such as gene mutations, chemotherapy and radiotherapy regimens, and relevant blood tumor markers was unavailable. For example, serum levels of D-dimer, neuron-specific enolase, and lactate dehydrogenase could be valuable biomarkers to predict survival in SCLC patients (Zhang et al. 2018; Liu et al. 2017). The absence of these data could have impacted the predictive efficacy of the nomogram. Second, the analysis did not include progression-free survival due to the limited information on patient outcomes in the SEER database. Third, despite the external validation being conducted, the generalizability of the nomogram to other populations needs further validation since the SEER database comprises only US populations.

Despite these limitations, this study is the first to develop a nomogram based on common and different prognostic factors to predict survival in ES-SCLC patients with organ-specific metastases. Additionally, including a larger sample size compared to previous related studies and the good predictive performance of the nomogram enhance the stability and reliability of the results. Therefore, the nomogram we constructed may serve as a useful tool for clinicians in predicting the prognosis of ES-SCLC patients with organ-specific metastasis.

## Conclusions

Patients with liver metastasis exhibited the poorest prognosis, while those with brain metastasis demonstrated the most favorable outcomes. Seven common factors, including older age, male sex, unmarried status, higher T stage, presence of other metastases, and receipt of radiotherapy and chemotherapy, were consistently associated with an unfavorable prognosis in ES-SCLC patients with metastases to the liver, lung, bone, and brain. Additionally, unique prognostic factors were identified: paired tumors for liver metastasis, lack of surgical treatment at the primary site for bone metastasis, and household income for brain metastasis. Using these factors, we constructed and validated four predictive nomograms, all demonstrating robust prediction performance. These nomograms serve as valuable clinical tools that help to accurately predict the prognosis of ES-SCLC patients with specific organ metastases.

## Supplementary Information

Below is the link to the electronic supplementary material.

Supplementary file1 (DOC 7509 KB)

## Data Availability

The data sets generated and/or analyzed during the current study are available in the SEER database (https://seer.cancer.gov/).
