# Supplementary material for: Prognostic factors in extensive-stage small cell lung cancer patients with organ-specific metastasis: unveiling commonalities and disparities
Source: J Cancer Res Clin Oncol. 2024 Feb 2;150(2):74. doi: 10.1007/s00432-024-05621-9 (PMC10837219; doi:10.1007/s00432-024-05621-9)

**SUPPORTING INFORMATION**

**Table S1.** Univariate and multivariate Cox regression analyses to evaluate prognostic factors for overall survival in ES-SCLC patients with liver metastasis.

**Table S2.** Univariate and multivariate Cox regression analyses to evaluate the prognostic factors for overall survival in ES-SCLC patients with lung metastasis.

**Table S3.** Univariate and multivariate Cox regression analyses to evaluate prognostic factors for overall survival in ES-SCLC patients with bone metastasis.

**Table S4.** Univariate and multivariate Cox regression analyses to evaluate the prognostic factors for overall survival in ES-SCLC patients with brain metastasis.

**Figure S1.** Kaplan–Meier curves of overall survival in ES-SCLC patients with different distant metastasis.

**Figure S2.** Calibration curves and the ROC curves of the nomogram to predict the 6-month, 1-year, and 2-year overall survival of ES-SCLC patients with liver metastasis. Calibration curves (a-c) and ROC curves (g-i) for the training cohort, and calibration curves (d-f) and ROC curves (j-l) for the validation cohort.

**Figure S3.** Calibration curves and the ROC curves of the nomogram to predict the 6-month, 1-year, and 2-year overall survival of ES-SCLC patients with lung metastasis. Calibration curves (a-c) and ROC curves (g-i) for the training cohort, and calibration curves (d-f) and ROC curves (j-l) for the validation cohort.

**Figure S4.** Calibration curves and the ROC curves of the nomogram to predict the 6-month, 1-year, and 2-year overall survival of ES-SCLC patients with bone metastasis. Calibration curves (a-c) and ROC curves (g-i) for the training cohort, and calibration curves (d-f) and ROC curves (j-l) for the validation cohort.

**Figure S5.** Calibration curves and the ROC curves of the nomogram to predict the 6-month, 1-year, and 2-year OS of ES-SCLC patients with brain metastasis. Calibration curves (a-c) and ROC curves (g-i) for the training cohort, and calibration curves (d-f) and ROC curves (j-l) for the validation cohort.

**Table S1.** Univariate and multivariable Cox regression analyses to evaluate the prognostic factors for overall survival in ES-SCLC patients with liver metastasis.

| **Subject characteristics** | **Univariate** | | | | **Multivariate** | | |
| --- | --- | --- | --- | --- | --- | --- | --- |
|  | **HR (95% CI)** | | **P-value** | | **HR (95% CI)** | | **P-value** |
| Age (years) |  | |  | |  | |  |
| ≤ 50 | 1(Reference) | | 1.00 | | 1(Reference) | | 1.00 |
| 51-60 | 1.07(0.95-1.22) | | 0.268 | | 1.03(0.91-1.17) | | 0.624 |
| 61-70 | 1.18(1.05-1.33) | | 0.007 | | 1.09(0.96-1.23) | | 0.168 |
| 71-80 | 1.40(1.24-1.58) | | ﹤0.001 | | 1.16(1.02-1.31) | | 0.020 |
| ≥81 | 1.94(1.70-2.21) | | ﹤0.001 | | 1.28(1.12-1.47) | | ﹤0.001 |
| Sex |  | |  | |  | |  |
| Female | 1(Reference) | | 1.00 | | 1(Reference) | | 1.00 |
| Male | 1.07(1.03-1.12) | | 0.001 | | 1.10(1.05-1.15) | | ﹤0.001 |
| Race |  | |  | |  | |  |
| White | 1(Reference) | | 1.00 | | 1(Reference) | | 1.00 |
| Black | 0.91(0.84-0.99) | | 0.023 | | 0.86(0.79-0.93) | | ﹤0.001 |
| Othersa | 0.90(0.81-1.00) | | 0.059 | | 0.86(0.77-0.97) | | 0.010 |
| Unknown | 1.30(0.72-2.35) | | 0.382 | | 1.20(0.66-2.17) | | 0.545 |
| Marital status |  | |  | |  | |  |
| Married | 1(Reference) | | 1.00 | | 1(Reference) | | 1.00 |
| Unmarriedb | 1.13(1.09-1.18) | | ﹤0.001 | | 1.09(1.04-1.13) | | ﹤0.001 |
| Unknown | 1.07(0.96-1.19) | | 0.257 | | 0.93(0.83-1.04) | | 0.197 |
| Household income |  | |  | |  | |  |
| ≤60,000$ | 1(Reference) | | 1.00 | | 1(Reference) | | 1.00 |
| ＞60,000$ | 0.96(0.92-1.00) | | 0.055 | | NA | | NA |
| Site |  |  | |  | |  | |
| Main bronchus | 1(Reference) | 1.00 | | 1(Reference) | | 1.00 | |
| Upper lobe | 0.99(0.92-1.06) | 0.677 | | 0.96(0.89-1.03) | | 0.226 | |
| Middle lobe | 0.98(0.86-1.11) | 0.709 | | 0.90(0.78-1.02) | | 0.107 | |
| Lower lobe | 1.02(0.94-1.10) | 0.673 | | 0.94(0.87-1.02) | | 0.114 | |
| Overlapping | 1.07(0.89-1.29) | 0.446 | | 0.92(0.77-1.11) | | 0.394 | |
| Unknown | 1.18(1.09-1.27) | ﹤0.001 | | 1.01(0.93-1.10) | | 0.848 | |
| Histological grade |  |  | |  | |  | |
| Grade I | 1(Reference) | 1.00 | | 1(Reference) | | 1.00 | |
| Grade II | 0.93(0.39-2.25) | 0.874 | | NA | | NA | |
| Grade III | 1.22(0.60-2.44) | 0.584 | | NA | | NA | |
| Grade IV | 1.25(0.62-2.51) | 0.528 | | NA | | NA | |
| Unknown | 1.29(0.65-2.58) | 0.469 | | NA | | NA | |
| Laterality |  |  | |  | |  | |
| Right | 1(Reference) | 1.00 | | 1(Reference) | | 1.00 | |
| Left | 1.01(0.97-1.06) | 0.557 | | 0.99(0.95-1.04) | | 0.696 | |
| Paired | 1.30(1.19-1.41) | ﹤0.001 | | 1.12(1.01-1.24) | | 0.033 | |
| Bilateral | 1.15(0.97-1.37) | 0.115 | | 1.07(0.89-1.28) | | 0.482 | |
| Others | 1.10(0.88-1.37) | 0.408 | | 1.07(0.86-1.34) | | 0.542 | |
| Lymphatic metastasis |  |  | |  | |  | |
| N0 | 1(Reference) | 1.00 | | 1(Reference) | | 1.00 | |
| N1 | 0.90(0.81-0.99) | 0.038 | | 0.97(0.88-1.08) | | 0.614 | |
| N2 | 0.92(0.86-0.99) | 0.028 | | 1.05(0.98-1.13) | | 0.144 | |
| N3 | 0.86(0.80-0.93) | ﹤0.001 | | 1.03(0.95-1.11) | | 0.477 | |
| Unknown | 1.13(1.02-1.25) | 0.020 | | 0.93(0.83-1.03) | | 0.162 | |
| T stage |  |  | |  | |  | |
| T1 | 1(Reference) | 1.00 | | 1(Reference) | | 1.00 | |
| T2 | 1.07(0.98-1.17) | 0.112 | | 1.10(1.01-1.20) | | 0.027 | |
| T3 | 1.13(1.04-1.23) | 0.006 | | 1.15(1.05-1.25) | | 0.002 | |
| T4 | 1.10(1.01-1.19) | 0.026 | | 1.12(1.03-1.22) | | 0.007 | |
| Unknown | 1.25(1.14-1.36) | 0.001 | | 1.09(0.99-1.19) | | 0.071 | |
| Lung metastasis |  |  | |  | |  | |
| None | 1(Reference) | 1.00 | | 1(Reference) | | 1.00 | |
| Yes | 1.11(1.05-1.17) | ﹤0.001 | | 1.06(1.00-1.12) | | 0.045 | |
| Unknown | 1.28(1.15-1.42) | ﹤0.001 | | 1.09(0.97-1.24) | | 0.153 | |
| Bone metastasis |  |  | |  | |  | |
| None | 1(Reference) | 1.00 | | 1(Reference) | | 1.00 | |
| Yes | 0.96(0.92-1.00) | 0.034 | | 1.05(1.01-1.10) | | 0.022 | |
| Unknown | 1.30(1.13-1.48) | ﹤0.001 | | 1.06(0.89-1.25) | | 0.516 | |
| Brain metastasis |  |  | |  | |  | |
| None | 1(Reference) | 1.00 | | 1(Reference) | | 1.00 | |
| Yes | 1.03(0.98-1.09) | 0.266 | | 1.26(1.19-1.34) | | ﹤0.001 | |
| Unknown | 1.29(1.14-1.45) | ﹤0.001 | | 0.99(0.85-1.15) | | 0.891 | |
| Surg(pri) |  |  | |  | |  | |
| Yes | 1(Reference) | 1.00 | | 1(Reference) | | 1.00 | |
| None | 1.40(1.00-1.96) | 0.051 | | NA | | NA | |
| Radiotherapy |  |  | |  | |  | |
| Yes | 1(Reference) | 1.00 | | 1(Reference) | | 1.00 | |
| No/unknown | 1.64(1.57-1.72) | ﹤0.001 | | 1.40(1.33-1.47) | | ﹤0.001 | |
| Chemotherapy |  |  | |  | |  | |
| Yes | 1(Reference) | 1.00 | | 1(Reference) | | 1.00 | |
| No/unknown | 3.90(3.72-4.09) | ﹤0.001 | | 3.61(3.43-3.79) | | ﹤0.001 | |

Abbreviations: NA=not available, Surg(pri)= surgical treatments of primary site.

a Includes American Indian/Alaska Native and Asian or Pacific Islander.

b Includes single, separated, widowed, and divorced.

**Table S2.** Univariate and multivariable Cox regression analyses to evaluate the prognostic factors for overall survival in ES-SCLC patients with lung metastasis.

| **Subject characteristics** | **Univariate** | | | | **Multivariate** | | |
| --- | --- | --- | --- | --- | --- | --- | --- |
|  | **HR (95% CI)** | | **P-value** | | **HR (95% CI)** | | **P-value** |
| Age (years) |  | |  | |  | |  |
| ≤ 50 | 1(Reference) | | 1.00 | | 1(Reference) | | 1.00 |
| 51-60 | 1.14(0.93-1.41) | | 0.213 | | 1.22(0.99-1.51) | | 0.063 |
| 61-70 | 1.29(1.06-1.59) | | 0.013 | | 1.31(1.07-1.61) | | 0.009 |
| 71-80 | 1.51(1.23-1.85) | | ﹤0.001 | | 1.51(1.23-1.85) | | 0.001 |
| ≥81 | 2.18(1.76-2.71) | | ﹤0.001 | | 1.68(1.34-2.09) | | ﹤0.001 |
| Sex |  | |  | |  | |  |
| Female | 1(Reference) | | 1.00 | | 1(Reference) | | 1.00 |
| Male | 1.14(1.07-1.22) | | ﹤0.001 | | 1.16(1.08-1.23) | | ﹤0.001 |
| Race |  | |  | |  | |  |
| White | 1(Reference) | | 1.00 | | 1(Reference) | | 1.00 |
| Black | 0.88(0.78-0.98) | | 0.021 | | 0.89(0.79-1.00) | | 0.046 |
| Othersa | 0.93(0.80-1.09) | | 0.376 | | 0.91(0.78-1.06) | | 0.236 |
| Unknown | 1.60(0.6-4.27) | | 0.346 | | 0.92(0.34-2.49) | | 0.877 |
| Marital status |  | |  | |  | |  |
| Married | 1(Reference) | | 1.00 | | 1(Reference) | | 1.00 |
| Unmarriedb | 1.15(1.07-1.22) | | ﹤0.001 | | 1.11(1.03-1.18) | | 0.003 |
| Unknown | 1.17(0.99-1.38) | | 0.064 | | 1.04(0.88-1.23) | | 0.640 |
| Household income |  | |  | |  | |  |
| ≤60,000$ | 1(Reference) | | 1.00 | | 1(Reference) | | 1.00 |
| ＞60,000$ | 0.96(0.90-1.02) | | 0.210 | | NA | | NA |
| Site |  |  | |  | |  | |
| Main bronchus | 1(Reference) | 1.00 | | 1(Reference) | | 1.00 | |
| Upper lobe | 0.98(0.88-1.09) | 0.715 | | 0.93(0.84-1.04) | | 0.203 | |
| Middle lobe | 0.98(0.8-1.21) | 0.872 | | 0.96(0.78-1.18) | | 0.712 | |
| Lower lobe | 1.06(0.94-1.2) | 0.323 | | 0.92(0.82-1.05) | | 0.211 | |
| Overlapping | 1.00(0.76-1.31) | 0.997 | | 1.06(0.81-1.39) | | 0.670 | |
| Unknown | 1.21(1.07-1.36) | 0.001 | | 1.05(0.92-1.19) | | 0.479 | |
| Histological grade |  |  | |  | |  | |
| Grade I | 1(Reference) | 1.00 | | 1(Reference) | | 1.00 | |
| Grade II | 0.84(0.23-3.10) | 0.791 | | NA | | NA | |
| Grade III | 1.26(0.41-3.94) | 0.685 | | NA | | NA | |
| Grade IV | 1.18(0.38-3.68) | 0.773 | | NA | | NA | |
| Unknown | 1.26(0.41-3.91) | 0.689 | | NA | | NA | |
| Laterality |  |  | |  | |  | |
| Right | 1(Reference) | 1.00 | | 1(Reference) | | 1.00 | |
| Left | 1.01(0.94-1.08) | 0.756 | | 1.02(0.95-1.10) | | 0.518 | |
| Paired | 1.28(1.11-1.47) | 0.001 | | 0.96(0.82-1.14) | | 0.655 | |
| Bilateral | 1.14(0.97-1.33) | 0.121 | | 0.97(0.82-1.16) | | 0.766 | |
| Others | 1.06(0.77-1.46) | 0.709 | | 1.07(0.78-1.49) | | 0.664 | |
| Lymphatic metastasis |  |  | |  | |  | |
| N0 | 1(Reference) | 1.00 | | 1(Reference) | | 1.00 | |
| N1 | 1.06(0.9-1.25) | 0.466 | | NA | | NA | |
| N2 | 1.10(0.98-1.23) | 0.110 | | NA | | NA | |
| N3 | 1.07(0.95-1.21) | 0.237 | | NA | | NA | |
| Unknown | 1.20(1.00-1.43) | 0.440 | | NA | | NA | |
| T stage |  |  | |  | |  | |
| T1 | 1(Reference) | 1.00 | | 1(Reference) | | 1.00 | |
| T2 | 1.11(0.92-1.34) | 0.286 | | 1.06(0.87-1.28) | | 0.568 | |
| T3 | 1.17(0.98-1.4) | 0.078 | | 1.17(0.98-1.40) | | 0.088 | |
| T4 | 1.17(0.99-1.39) | 0.069 | | 1.21(1.02-1.44) | | 0.030 | |
| Unknown | 1.44(1.19-1.75) | ﹤0.001 | | 1.27(1.04-1.54) | | 0.019 | |
| Liver metastasis |  |  | |  | |  | |
| None | 1(Reference) | 1.00 | | 1(Reference) | | 1.00 | |
| Yes | 1.44(1.35-1.54) | ﹤0.001 | | 1.40(1.31-1.51) | | ﹤0.001 | |
| Unknown | 1.25(1.00-1.56) | 0.045 | | 0.96(0.73-1.28) | | 0.796 | |
| Bone metastasis |  |  | |  | |  | |
| None | 1(Reference) | 1.00 | | 1(Reference) | | 1.00 | |
| Yes | 1.17(1.10-1.25) | ﹤0.001 | | 1.17(1.09-1.26) | | ﹤0.001 | |
| Unknown | 1.23(1.01-1.5) | 0.036 | | 1.03(0.79-1.33) | | 0.831 | |
| Brain metastasis |  |  | |  | |  | |
| None | 1(Reference) | 1.00 | | 1(Reference) | | 1.00 | |
| Yes | 1.09(1.00-1.17) | 0.038 | | 1.40(1.29-1.52) | | ﹤0.001 | |
| Unknown | 1.30(1.09-1.55) | 0.004 | | 0.94(0.74-1.19) | | 0.600 | |
| Surg(pri) |  |  | |  | |  | |
| Yes | 1(Reference) | 1.00 | | 1(Reference) | | 1.00 | |
| None | 1.37(0.91-2.06) | 0.136 | | NA | | NA | |
| Radiotherapy |  |  | |  | |  | |
| Yes | 1(Reference) | 1.00 | | 1(Reference) | | 1.00 | |
| No/unknown | 1.75(1.64-1.88) | ﹤0.001 | | 1.46(1.36-1.58) | | ﹤0.001 | |
| Chemotherapy |  |  | |  | |  | |
| Yes | 1(Reference) | 1.00 | | 1(Reference) | | 1.00 | |
| No/unknown | 3.47(3.23-3.72) | ﹤0.001 | | 3.32(3.08-3.59) | | ﹤0.001 | |

Abbreviations: NA=not available, Surg(pri)= surgical treatments of primary site.

a Includes American Indian/Alaska Native and Asian or Pacific Islander.

b Includes single, separated, widowed, and divorced.

**Table S3.** Univariate and multivariable Cox regression analyses to evaluate the prognostic factors for overall survival in ES-SCLC patients with bone metastasis.

| **Subject characteristics** | **Univariate** | | | | **Multivariate** | | |
| --- | --- | --- | --- | --- | --- | --- | --- |
|  | **HR (95% CI)** | | **P-value** | | **HR (95% CI)** | | **P-value** |
| Age (years) |  | |  | |  | |  |
| ≤ 50 | 1(Reference) | | 1.00 | | 1(Reference) | | 1.00 |
| 51-60 | 1.01(0.87-1.16) | | 0.915 | | 1.01(0.87-1.16) | | 0.907 |
| 61-70 | 1.14(0.99-1.31) | | 0.061 | | 1.13(0.98-1.29) | | 0.091 |
| 71-80 | 1.39(1.21-1.60) | | ﹤0.001 | | 1.27(1.11-1.46) | | 0.001 |
| ≥81 | 1.86(1.60-2.17) | | ﹤0.001 | | 1.49(1.28-1.75) | | ﹤0.001 |
| Sex |  | |  | |  | |  |
| Female | 1(Reference) | | 1.00 | | 1(Reference) | | 1.00 |
| Male | 1.11(1.06-1.17) | | ﹤0.001 | | 1.17(1.11-1.23) | | ﹤0.001 |
| Race |  | |  | |  | |  |
| White | 1(Reference) | | 1.00 | | 1(Reference) | | 1.00 |
| Black | 0.94(0.86-1.03) | | 0.192 | | NA | | NA |
| Othersa | 0.98(0.86-1.11) | | 0.722 | | NA | | NA |
| Unknown | 0.70(0.23-2.18) | | 0.544 | | NA | | NA |
| Marital status |  | |  | |  | |  |
| Married | 1(Reference) | | 1.00 | | 1(Reference) | | 1.00 |
| Unmarriedb | 1.18(1.13-1.24) | | ﹤0.001 | | 1.18(1.12-1.24) | | ﹤0.001 |
| Unknown | 1.02(0.90-1.17) | | 0.715 | | 1.00(0.87-1.14) | | 0.962 |
| Household income |  | |  | |  | |  |
| ≤60,000$ | 1(Reference) | | 1.00 | | 1(Reference) | | 1.00 |
| ＞60,000$ | 0.98(0.94-1.03) | | 0.461 | | NA | | NA |
| Site |  |  | |  | |  | |
| Main bronchus | 1(Reference) | 1.00 | | 1(Reference) | | 1.00 | |
| Upper lobe | 1.01(0.93-1.10) | 0.759 | | 0.99(0.91-1.07) | | 0.788 | |
| Middle lobe | 0.88(0.75-1.02) | 0.094 | | 0.86(0.74-1.01) | | 0.060 | |
| Lower lobe | 1.07(0.98-1.17) | 0.151 | | 1.03(0.94-1.13) | | 0.526 | |
| Overlapping | 1.06(0.86-1.30) | 0.601 | | 1.06(0.86-1.3) | | 0.611 | |
| Unknown | 1.15(1.05-1.26) | 0.004 | | 1.02(0.93-1.13) | | 0.645 | |
| Histological grade |  |  | |  | |  | |
| Grade I | 1(Reference) | 1.00 | | 1(Reference) | | 1.00 | |
| Grade II | 1.96(0.76-5.08) | 0.166 | | NA | | NA | |
| Grade III | 1.75(0.87-3.52) | 0.117 | | NA | | NA | |
| Grade IV | 1.79(0.89-3.59) | 0.103 | | NA | | NA | |
| Unknown | 1.79(0.90-3.59) | 0.099 | | NA | | NA | |
| Laterality |  |  | |  | |  | |
| Right | 1(Reference) | 1.00 | | 1(Reference) | | .158 | |
| Left | 1.00(0.95-1.05) | 0.984 | | 0.96(0.91-1.01) | | 0.682 | |
| Paired | 1.30(1.16-1.45) | ﹤0.001 | | 1.03(0.9-1.17) | | 0.271 | |
| Bilateral | 1.22(0.98-1.51) | 0.073 | | 1.13(0.91-1.42) | | 0.932 | |
| Others | 1.03(0.80-1.34) | 0.802 | | 0.99(0.76-1.29) | | 0.158 | |
| Lymphatic metastasis |  |  | |  | |  | |
| N0 | 1(Reference) | 1.00 | | 1(Reference) | | 1.00 | |
| N1 | 1.03(0.91-1.17) | 0.635 | | 1.08(0.95-1.23) | | 0.231 | |
| N2 | 1.01(0.93-1.10) | 0.793 | | 1.14(1.04-1.24) | | 0.005 | |
| N3 | 0.97(0.88-1.06) | 0.485 | | 1.11(1.01-1.23) | | 0.025 | |
| Unknown | 1.23(1.08-1.40) | 0.002 | | 1.04(0.90-1.19) | | 0.597 | |
| T stage |  |  | |  | |  | |
| T1 | 1(Reference) | 1.00 | | 1(Reference) | | 1.00 | |
| T2 | 1.09(0.99-1.21) | 0.077 | | 1.07(0.97-1.18) | | 0.203 | |
| T3 | 1.10(0.99-1.21) | 0.068 | | 1.09(0.99-1.21) | | 0.092 | |
| T4 | 1.13(1.03-1.24) | 0.008 | | 1.16(1.06-1.28) | | 0.002 | |
| Unknown | 1.32(1.19-1.46) | ﹤0.001 | | 1.17(1.05-1.30) | | 0.005 | |
| Liver metastasis |  |  | |  | |  | |
| None | 1(Reference) | 1.00 | | 1(Reference) | | 1.00 | |
| Yes | 1.38(1.32-1.45) | ﹤0.001 | | 1.36(1.29-1.43) | | ﹤0.001 | |
| Unknown | 1.27(1.04-1.54) | 0.019 | | 1.05(0.84-1.31) | | 0.682 | |
| Lung metastasis |  |  | |  | |  | |
| None | 1(Reference) | 1.00 | | 1(Reference) | | 1.00 | |
| Yes | 1.25(1.18-1.33) | ﹤0.001 | | 1.12(1.05-1.19) | | 0.001 | |
| Unknown | 1.15(1.01-1.31) | 0.030 | | 0.87(0.75-1.01) | | 0.066 | |
| Brain metastasis |  |  | |  | |  | |
| None | 1(Reference) | 1.00 | | 1(Reference) | | 1.00 | |
| Yes | 1.15(1.08-1.22) | ﹤0.001 | | 1.27(1.19-1.36) | | ﹤0.001 | |
| Unknown | 1.41(1.22-1.64) | ﹤0.001 | | 1.05(0.89-1.25) | | 0.538 | |
| Surg(pri) |  |  | |  | |  | |
| Yes | 1(Reference) | 1.00 | | 1(Reference) | | 1.00 | |
| None | 1.60(1.11-2.31) | 0.011 | | 1.57(1.09-2.26) | | 0.016 | |
| Radiotherapy |  |  | |  | |  | |
| Yes | 1(Reference) | 1.00 | | 1(Reference) | | 1.00 | |
| No/unknown | 1.52(1.44-1.60) | ﹤0.001 | | 1.30(1.23-1.37) | | ﹤0.001 | |
| Chemotherapy |  |  | |  | |  | |
| Yes | 1(Reference) | 1.00 | | 1(Reference) | | 1.00 | |
| No/unknown | 4.05(3.82-4.29) | ﹤0.001 | | 3.77(3.54-4.00) | | ﹤0.001 | |

Abbreviations: NA=not available, Surg(pri)= surgical treatments of primary site.

a Includes American Indian/Alaska Native and Asian or Pacific Islander.

b Includes single, separated, widowed, and divorced.

**Table S4.** Univariate and multivariable Cox regression analyses to evaluate the prognostic factors for overall survival in ES-SCLC patients with brain metastasis.

| **Subject characteristics** | **Univariate** | | | | **Multivariate** | | |
| --- | --- | --- | --- | --- | --- | --- | --- |
|  | **HR (95% CI)** | | **P-value** | | **HR (95% CI)** | | **P-value** |
| Age (years) |  | |  | |  | |  |
| ≤ 50 | 1(Reference) | | 1.00 | | 1(Reference) | | 1.00 |
| 51-60 | 1.15(0.98-1.35) | | 0.092 | | 1.15(0.97-1.35) | | 0.102 |
| 61-70 | 1.20(1.03-1.41) | | 0.023 | | 1.16(0.99-1.36) | | 0.070 |
| 71-80 | 1.61(1.37-1.89) | | ﹤0.001 | | 1.50(1.28-1.76) | | ﹤0.001 |
| ≥81 | 2.46(2.04-2.96) | | ﹤0.001 | | 1.69(1.39-2.04) | | ﹤0.001 |
| Sex |  | |  | |  | |  |
| Female | 1(Reference) | | 1.00 | | 1(Reference) | | 1.00 |
| Male | 1.13(1.07-1.20) | | ﹤0.001 | | 1.16(1.09-1.23) | | ﹤0.001 |
| Race |  | |  | |  | |  |
| White | 1(Reference) | | 1.00 | | 1(Reference) | | 1.00 |
| Black | 1.00(0.91-1.10) | | 0.955 | | NA | | NA |
| Othersa | 0.90(0.79-1.02) | | 0.103 | | NA | | NA |
| Unknown | 1.24(0.56-2.76) | | 0.602 | | NA | | NA |
| Marital status |  | |  | |  | |  |
| Married | 1(Reference) | | 1.00 | | 1(Reference) | | 1.00 |
| Unmarriedb | 1.14(1.08-1.21) | | ﹤0.001 | | 1.10(1.04-1.17) | | 0.002 |
| Unknown | 1.14(0.98-1.33) | | 0.097 | | 1.04(0.89-1.21) | | 0.655 |
| Household income |  | |  | |  | |  |
| ≤60,000$ | 1(Reference) | | 1.00 | | 1(Reference) | | 1.00 |
| ＞60,000$ | 0.89(0.84-0.94) | | ﹤0.001 | | 0.86(0.81-0.91) | | ﹤0.001 |
| Site |  |  | |  | |  | |
| Main bronchus | 1(Reference) | 1.00 | | 1(Reference) | | 1.00 | |
| Upper lobe | 0.94(0.85-1.04) | 0.233 | | NA | | NA | |
| Middle lobe | 0.87(0.73-1.04) | 0.129 | | NA | | NA | |
| Lower lobe | 1.06(0.95-1.18) | 0.317 | | NA | | NA | |
| Overlapping | 0.80(0.60-1.06) | 0.122 | | NA | | NA | |
| Unknown | 1.06(0.95-1.19) | 0.291 | | NA | | NA | |
| Histological grade |  |  | |  | |  | |
| Grade I | 1(Reference) | 1.00 | | 1(Reference) | | 1.00 | |
| Grade II | 2.11(0.80-5.56) | 0.130 | | NA | | NA | |
| Grade III | 2.14(0.95-4.79) | 0.065 | | NA | | NA | |
| Grade IV | 2.02(0.90-4.53) | 0.086 | | NA | | NA | |
| Unknown | 2.16(0.97-4.8) | 0.060 | | NA | | NA | |
| Laterality |  |  | |  | |  | |
| Right | 1(Reference) | 1.00 | | 1(Reference) | | 1.00 | |
| Left | 1.05(0.99-1.12) | 0.086 | | 1.02(0.96-1.09) | | 0.285 | |
| Paired | 1.06(0.94-1.21) | 0.339 | | 0.93(0.81-1.06) | | 0.811 | |
| Bilateral | 1.43(1.09-1.88) | 0.010 | | 1.03(0.78-1.36) | | 0.356 | |
| Others | 1.18(0.88-1.57) | 0.264 | | 1.15(0.86-1.54) | | 0.285 | |
| Lymphatic metastasis |  |  | |  | |  | |
| N0 | 1(Reference) | 1.00 | | 1(Reference) | | 1.00 | |
| N1 | 0.98(0.86-1.12) | 0.794 | | 1.07(0.94-1.21) | | 0.332 | |
| N2 | 1.18(1.07-1.28) | ﹤0.001 | | 1.29(1.18-1.42) | | ﹤0.001 | |
| N3 | 1.21(1.10-1.34) | ﹤0.001 | | 1.33(1.20-1.47) | | ﹤0.001 | |
| Unknown | 1.26(1.09-1.45) | 0.001 | | 1.13(0.97-1.31) | | 0.112 | |
| T stage |  |  | |  | |  | |
| T1 | 1(Reference) | 1.00 | | 1(Reference) | | 1.00 | |
| T2 | 1.08(0.97-1.2) | 0.179 | | 1.07(0.95-1.19) | | 0.255 | |
| T3 | 1.19(1.07-1.34) | 0.002 | | 1.16(1.03-1.30) | | 0.012 | |
| T4 | 1.17(1.05-1.29) | 0.004 | | 1.16(1.04-1.28) | | 0.007 | |
| Unknown | 1.13(1.01-1.27) | 0.033 | | 1.04(0.92-1.18) | | 0.535 | |
| Liver metastasis |  |  | |  | |  | |
| None | 1(Reference) | 1.00 | | 1(Reference) | | 1.00 | |
| Yes | 1.56(1.46-1.66) | ﹤0.001 | | 1.48(1.38-1.58) | | ﹤0.001 | |
| Unknown | 1.32(1.07-1.62) | 0.009 | | 0.99(0.77-1.27) | | 0.928 | |
| Lung metastasis |  |  | |  | |  | |
| None | 1(Reference) | 1.00 | | 1(Reference) | | 1.00 | |
| Yes | 1.25(1.16-1.35) | ﹤0.001 | | 1.09(1.00-1.18) | | 0.041 | |
| Unknown | 1.23(1.05-1.45) | 0.009 | | 0.92(0.76-1.11) | | 0.377 | |
| Bone metastasis |  |  | |  | |  | |
| None | 1(Reference) | 1.00 | | 1(Reference) | | 1.00 | |
| Yes | 1.30(1.22-1.38) | ﹤0.001 | | 1.16(1.08-1.24) | | ﹤0.001 | |
| Unknown | 1.57(1.24-2.01) | ﹤0.001 | | 1.35(1.02-1.79) | | 0.036 | |
| Surg(pri) |  |  | |  | |  | |
| Yes | 1(Reference) | 1.00 | | 1(Reference) | | 1.00 | |
| None | 1.67(1.23-2.29) | 0.001 | | 1.32(0.96-1.80) | | 0.084 | |
| Radiotherapy |  |  | |  | |  | |
| Yes | 1(Reference) | 1.00 | | 1(Reference) | | 1.00 | |
| No/unknown | 1.80(1.69-1.91) | ﹤0.001 | | 1.36(1.27-1.45) | | ﹤0.001 | |
| Chemotherapy |  |  | |  | |  | |
| Yes | 1(Reference) | 1.00 | | 1(Reference) | | 1.00 | |
| No/unknown | 3.27(3.06-3.49) | ﹤0.001 | | 3.20(2.98-3.43) | | ﹤0.001 | |

Abbreviations: NA=not available, Surg(pri)= surgical treatments of primary site.

a Includes American Indian/Alaska Native and Asian or Pacific Islander.

b Includes single, separated, widowed, and divorced.

**Figure S1.** Kaplan–Meier curves of overall survival in ES-SCLC patients with different distant metastasis.


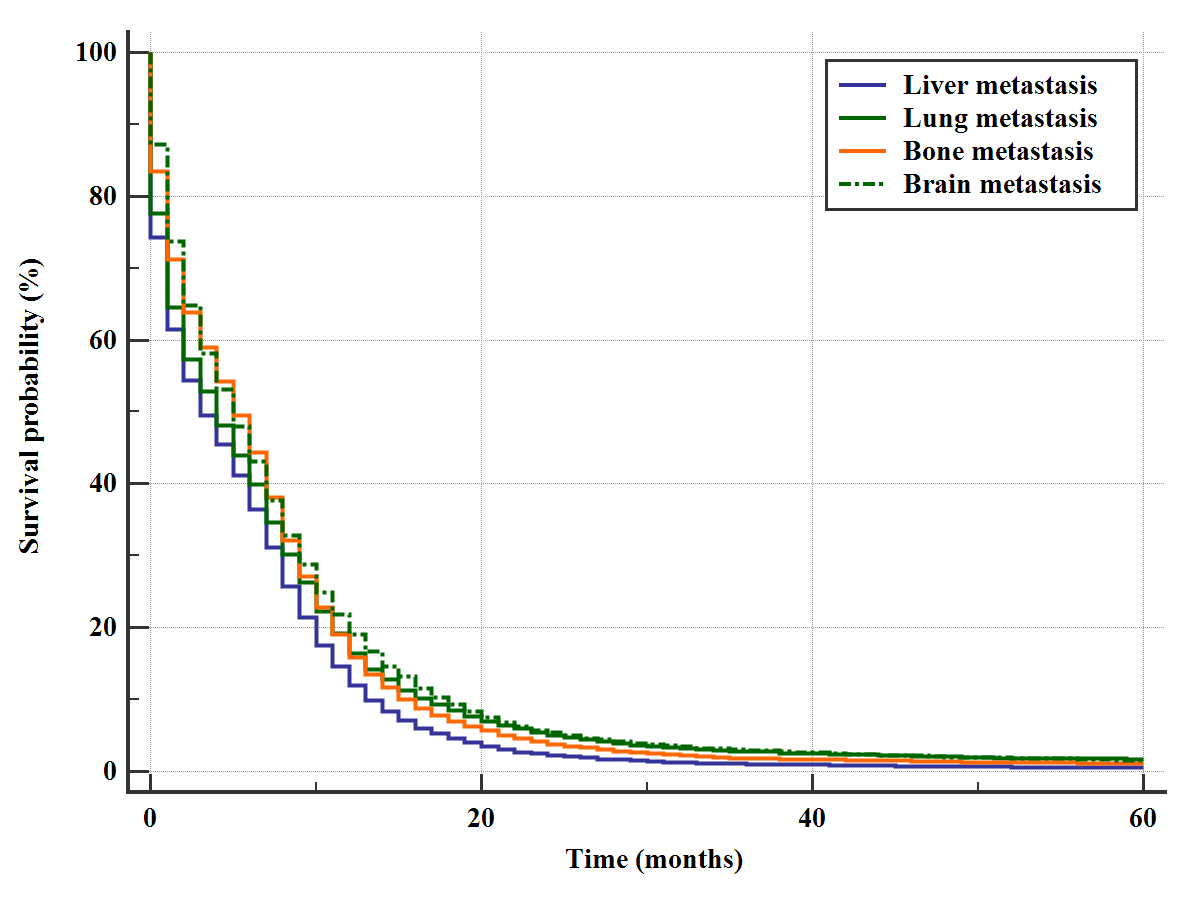


**Figure S2.** Calibration curves and ROC curves of nomogram for predicting the 6-month, 1-year, and 2-year overall survival of ES-SCLC patients with liver metastasis. Calibration curves (a-c) and ROC curves (g-i) for the training cohort, and calibration curves (d-f) and ROC curves (j-l) for the validation cohort.


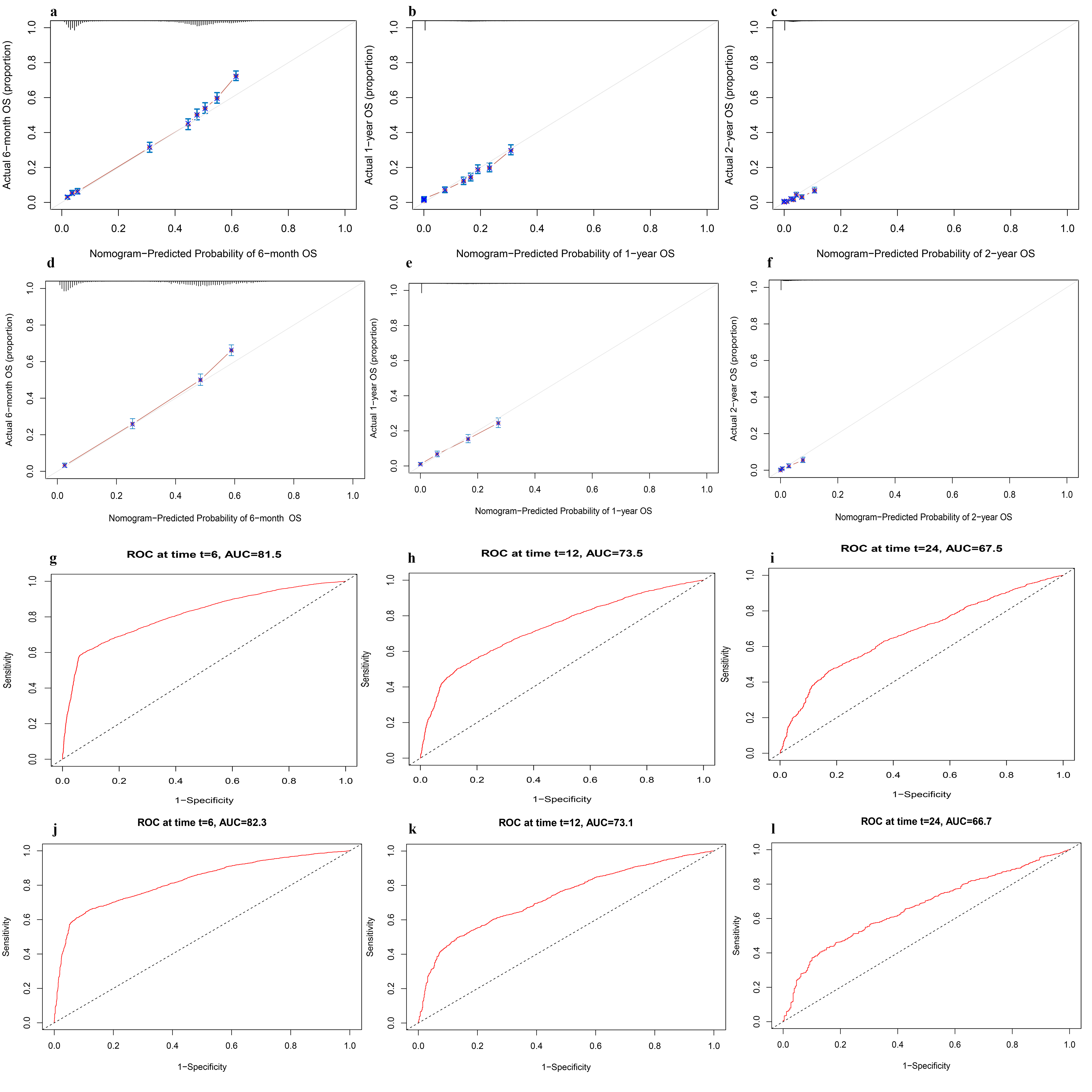


**Figure S3.** Calibration curves and ROC curves of the nomogram for predicting the 6-month, 1-year, and 2-year overall survival of ES-SCLC patients with lung metastasis. Calibration curves (a-c) and ROC curves (g-i) for the training cohort, and calibration curves (d-f) and ROC curves (j-l) for the validation cohort.


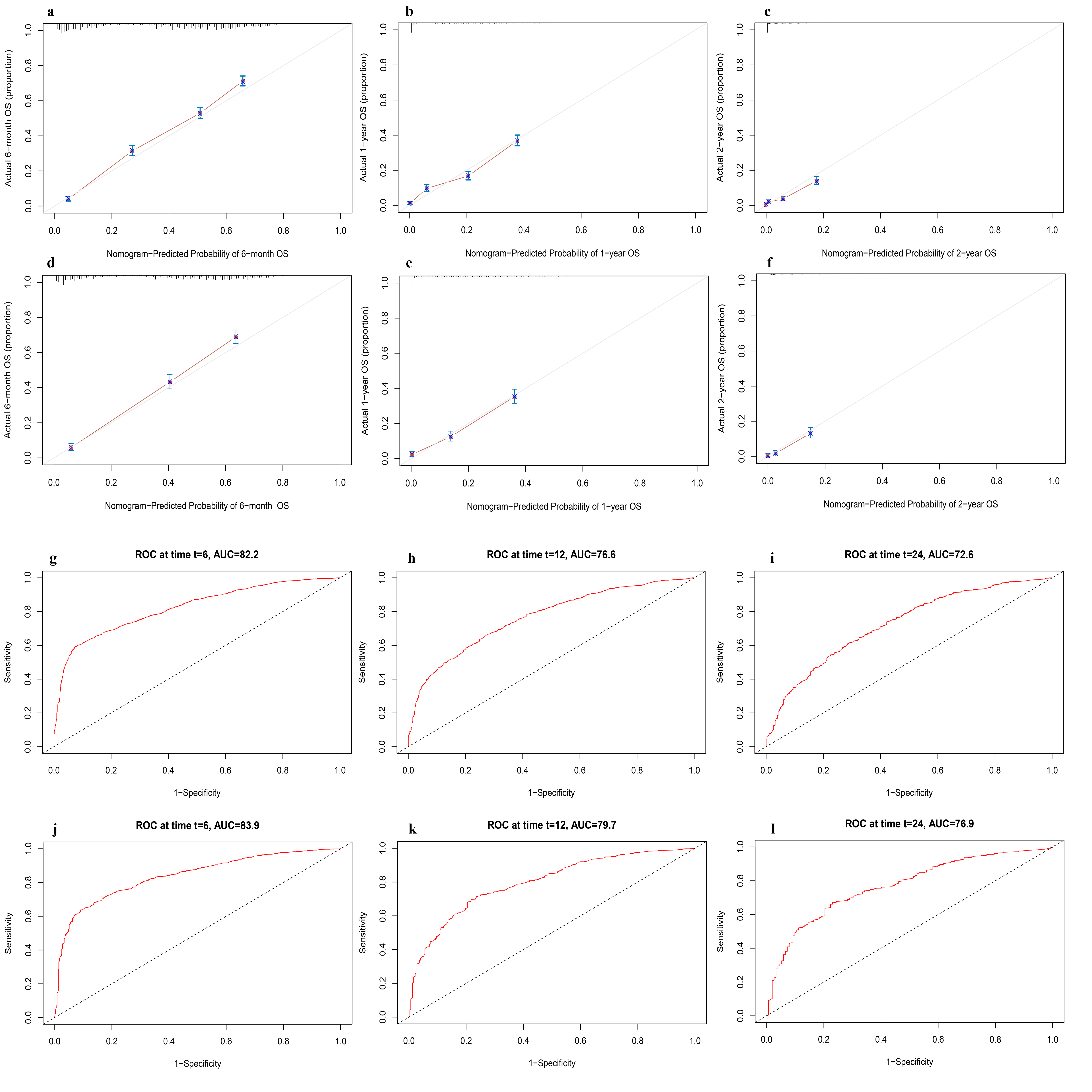


**Figure S4.** Calibration curves and ROC curves of the nomogram for predicting the 6-month, 1-year, and 2-year overall survival of ES-SCLC patients with bone metastasis. Calibration curves (a-c) and ROC curves (g-i) for the training cohort, and calibration curves (d-f) and ROC curves (j-l) for the validation cohort.


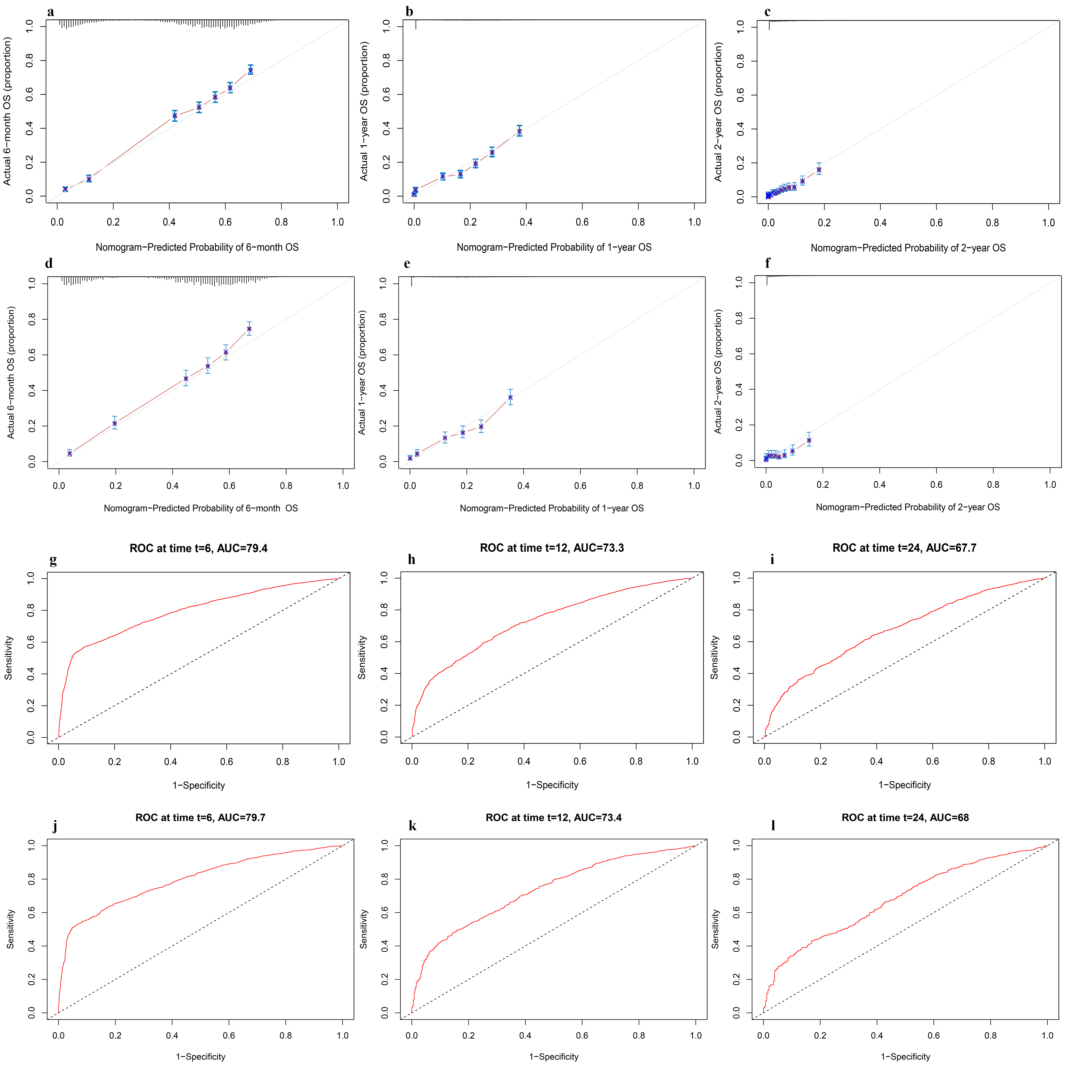


**Figure S5.** Calibration curves and ROC curves of the nomogram for predicting the 6-month, 1-year, and 2-year overall survival of ES-SCLC patients with brain metastasis. Calibration curves (a-c) and ROC curves (g-i) for the training cohort, and calibration curves (d-f) and ROC curves (j-l) for the validation cohort.


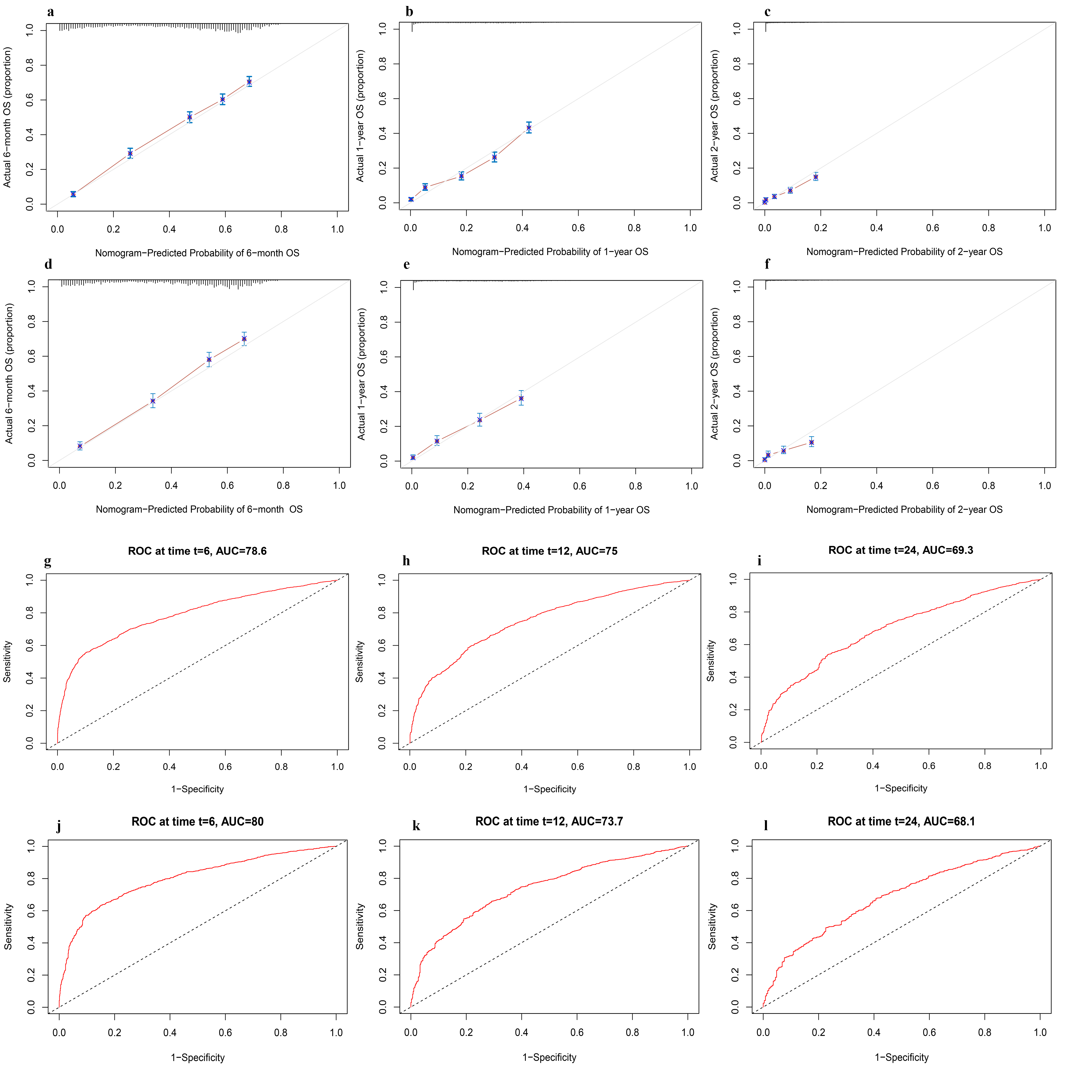

Supplement: Supplementary file 1 — Supplementary file1 (DOC 7509 KB) [file 432_2024_5621_MOESM1_ESM.doc]
